# The C‐terminal cytosolic domain of the human zinc transporter ZnT8 and its diabetes risk variant

**DOI:** 10.1111/febs.14402

**Published:** 2018-02-27

**Authors:** Douglas S. Parsons, Christer Hogstrand, Wolfgang Maret

**Affiliations:** ^1^ Metal Metabolism Group Departments of Biochemistry and Nutritional Sciences Faculty of Life Sciences and Medicine King's College London UK

**Keywords:** cation diffusion facilitator, C‐terminal domain, diabetes, zinc, ZnT8

## Abstract

A significant aspect of the control of cellular zinc in eukarya is its subcellular re‐distribution. One of the four human vesicular zinc transporters, ZnT8, supplies the millimolar zinc concentrations of insulin granules in pancreatic β‐cells, affecting insulin processing, crystallisation and secretion. ZnT8 has a transmembrane and a C‐terminal cytosolic domain; the latter has important functions and purportedly mediates protein–protein interactions, senses cytosolic zinc and/or channels zinc to the transport site in the transmembrane domain (TMD). A common variant W325R in the C‐terminal domain (CTD) increases the risk to develop type 2 diabetes and affects autoantibody specificity in type 1 diabetes. To investigate the differences between the two protein variants, we purified and biophysically characterised both variants of the ZnT8 CTD [R325 variant of ZnT8 CTD (aa267–369) (ZnT8cR) and W325 variant of ZnT8 CTD (aa267–369) (ZnT8cW)]. The domains fold independently of the TMD. Remarkably, the ZnT8cW variant (diabetes protection in the full‐length protein) is less thermostable than the ZnT8cR variant (diabetes risk in the full‐length protein). The ZnT8cW monomers associate with higher affinity. Both CTD variants bind zinc with a stoichiometry that differs from bacterial homologues, emphasising the limitation of the latter as models for the structure and function of the human proteins. The relatively small but reproducible differences between the two ZnT8 CTD variants begin to provide a molecular basis for the different diabetes susceptibility caused by the full‐length ZnT8 proteins.

**Abbreviations**
Ch. Int.: charge interlock
CTD: C‐terminal domain
ICP‐MS: inductively coupled plasma mass spectrometry
MST: microscale thermophoresis
nDSF: nano differential scanning fluorimetry
T1D: type 1 diabetes
T2D: type 2 diabetes
TMD: transmembrane domain
ZnT8cR: R325 variant of ZnT8 CTD (aa267‐369)
ZnT8cW: W325 variant of ZnT8 CTD (aa267–369)
ZnT: zinc transporter

## Introduction

Perturbations of cellular zinc metabolism through mutations in dozens of proteins are implicated in a growing number of human diseases 1. Among these proteins are transporters that regulate the passage of Zn^2+^ across biological membranes. Belonging to two families, ZIPs (Zrt/Irt‐like Protein, *SLC39A*) and ZnTs (zinc transporter, *SLC30A*), they transport zinc into and out of the cytosol, respectively 2. Humans have 14 ZIPs and 10 ZnTs, which are expressed differentially in tissues and have different cellular localisations 2.

Cellular zinc compartmentalisation is controlled by these two families of zinc transporters and by metallothioneins 3. A subgroup of four transporters (ZnT2, 3, 4 and 8 in mammals) of the cation diffusion facilitator (CDF) superfamily exports zinc ions from the cytosol into intracellular vesicles. One of them, ZnT8 (*SLC30A8*), is highly expressed in the membrane of the dense insulin secretory granules of pancreatic β‐cells 4. It supplies zinc for the storage of insulin as a crystalline hexamer and for other aspects of the biochemistry occurring in the granules 5. ZnT8 also has a yet to be defined role in glucagon secretion in pancreatic α‐cells and is present in some other tissues 6.

A structural issue of ZnT8 with significant functional implications is an SNP resulting in two major variants with either Arg (R) or Trp (W) at position 325 in the C‐terminal domain (CTD). Both variants are common in human populations (R: 60%–95%; W: 5%–40%) 7. A third variant encoding Gln is far rarer in populations and will not be discussed here. Remarkably, the major R325 variant increases the risk of developing type 2 diabetes (T2D) 5. These observations have led to the postulate that higher zinc transport activity is related to β‐cell health and that activation of ZnT8 might be a way of improving β‐cell health 8. However, this interpretation has now come into question, as the R325 T2D‐risk allele apparently has the higher transport activity 9, providing a plausible explanation why humans heterozygous for one of several loss‐of‐function mutations in *SLC30A8* are protected against diabetes 10. Based on 3D homology models, the R/W325 replacement in ZnT8 is not directly involved in the CTD zinc‐binding site(s). However, it has functional consequences and a relatively large impact on human health, as the differences in transport activity of the two variants confer a 15% increased risk of developing T2D for each allele of the risk R325 form 9, 11.

There is no 3D structure of a eukaryotic CDF protein; therefore, concepts of how these vesicular ZnTs work are based entirely on the 3D structures of prokaryotic CDF homologues. The crystal structure of the *Escherichia coli* protein YiiP revealed a dimer with a transmembrane domain (TMD) and a CTD 12. The dimer binds eight zinc ions: two in sites A, which are the primary TMD transport sites, two in sites B between the domains with an unknown function and four in two binuclear sites C at the dimer interface of the CTD. The CTDs are believed to be metal sensors, working by an allosteric mechanism that links the occupancy of sites C by zinc (*K*_d_ = 24 μm, measured in proteoliposomes) to induction of a conformational change of the CTD, which is then transmitted to the TMD to trigger zinc/proton antiport 13. This allosteric regulation of the TMD by the CTD is thought to be important for full transporter functionality, as though CTD‐truncated versions of the CDF proteins ZitB from *E. coli* and CzcD from *Cupriavidus metallidurans* were capable of transporting Zn^2+^ *in vivo*, their transport activity was decreased 14. However, mutations at site C in the CTD of MntE from *Streptococcus pneumoniae* did not affect manganese transport 15. Additionally, a cryo‐electron microscopy structure of a YiiP homologue from *Shewanella oneidensis* does not seem to confirm the allosteric mechanism of zinc binding; rather this study suggests that the zinc‐binding sites in the CTD are of such high affinity that zinc is always bound; therefore, any conformational changes during transport occur solely in the TMD 16. These data suggest that different CDF CTDs have varying effects on transport function even among bacterial homologues.

Aside from the structure of the full‐length *E. coli* protein YiiP, there are three crystal structures of bacterial CTDs: *Thermus thermophilus* CzrB 17, *Thermotoga maritima* TM0876 18 and *Magnetospirillum gryphiswaldense* MamM 19. The models of the bacterial proteins fail to explain the function(s) of the CTD in the family of mammalian vesicular transporters for the following reason. While the bacterial proteins sense and export an excess of zinc, there is no evidence for an excess of zinc in the cytosol of eukaryotic cells for export into granules of the secretory pathway. Unless zinc is made available by some yet unknown mechanism, the cytosolic free zinc ion concentrations are only hundreds of pm to maximally 1.5 nm, a very small fraction of the ~ 250 μm total cellular zinc concentration 20, 21. In insulin granules, estimates of free zinc ion concentrations are 120 nm (pH 6) and total zinc concentrations are tens to perhaps even hundreds of millimolar 22, 23. Thus, for both total and free zinc, ZnT8 has to work against a concentration gradient of about three orders of magnitude.

The R325W replacement in ZnT8 generates a different epitope for autoantibodies in type 1 diabetes (T1D) 24 (an issue of protein conformation in the CTD) in addition to affecting insulin biology in T2D (an issue thought to relate to zinc transport) 9. The underlying question for the basic biological chemistry addressed here is how these two amino acids affect subunit interactions, dimer dynamics and zinc binding. Thus, biophysical investigations of the CTDs of ZnT8 would solve a key issue in β‐cell granule biology central to control of energy metabolism, provide important information regarding the biology of other zinc‐containing vesicles served by ZnT2–4, and make a significant contribution to CDF biology in general. Towards this goal, we expressed both ZnT8cW (ZnT8 CTD, aa267–369, expressing Trp at position 325) and ZnT8cR (ZnT8 CTD expressing Arg at position 325). The two proteins adopt their predicted fold independent of the presence of the TMD and have different zinc‐binding characteristics compared to their bacterial homologues. Structural and stability differences between the two CTD variants affect their dimerisation. Previous deductions made from the 3D structures of bacterial homologues are therefore insufficient to explain the properties of the human proteins in health and disease.

## Results

### Bioinformatics structure predictions

**Figure 1:**
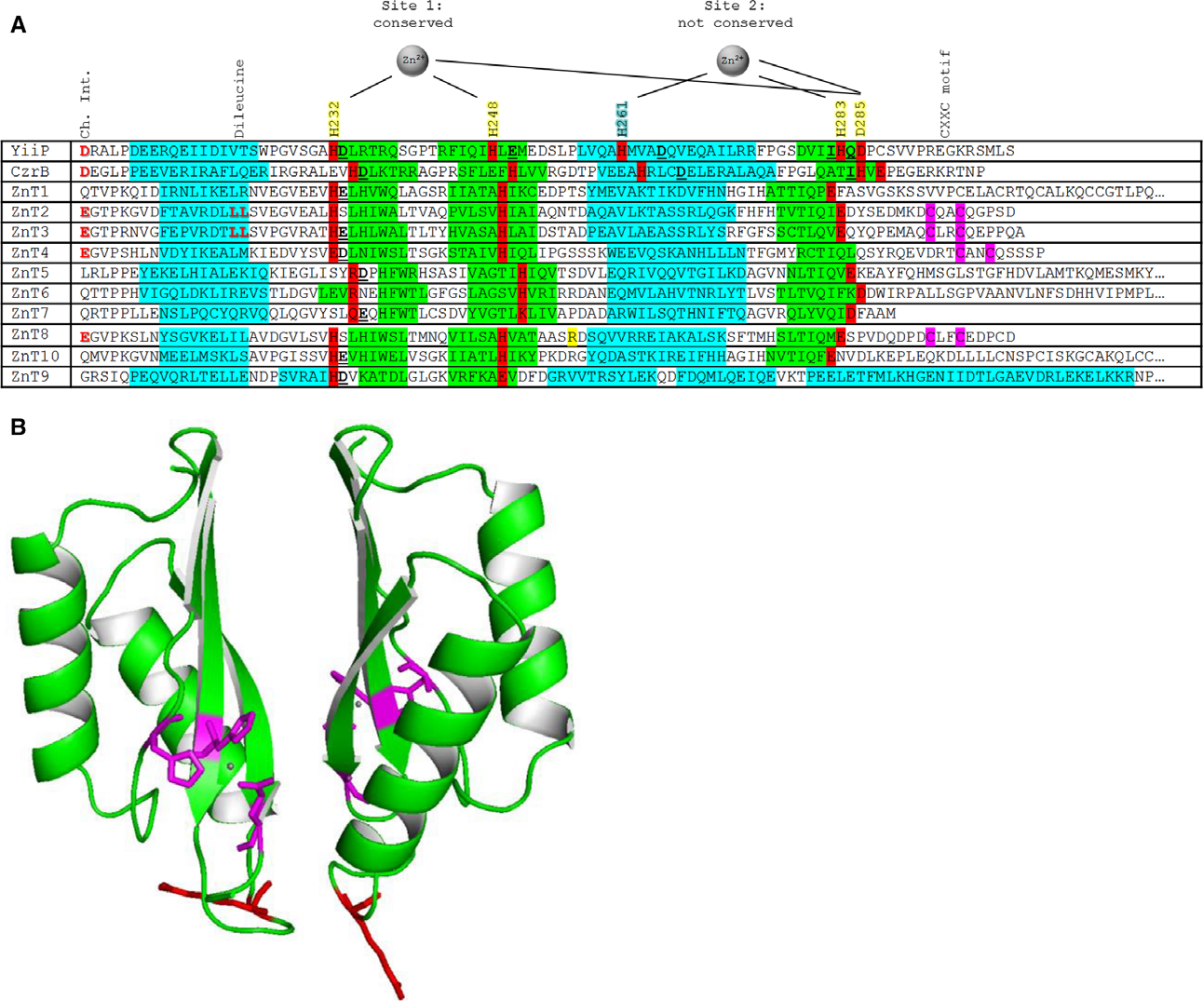
Metal‐binding and structural motifs in the CTDs of ZnTs/CDFs. (A) Primary sequence comparison between the CTDs of selected bacterial and human CDFs, indicating both conserved and non‐conserved motifs. Protein secondary structure was predicted using jpred 4 (Materials and methods); α‐helices in blue and β‐sheets in green. Metal‐binding residues are highlighted in red with black text; site 2 of the binuclear zinc site described in the 3D structure of *Escherichia coli* YiiP (shown on top) is not conserved in mammalian ZnTs. Metal‐binding residues annotated in the alignment are contributed from one protomer (yellow) or the other protomer (blue) in the dimer. Both metal‐binding sites in *E. coli* YiiP utilise a water molecule as the fourth ligand in the tetrahedral coordination of each Zn^2+^ ion. Specific residue numbering is based on the sequence of the *E. coli* YiiP protein. The arginine at position 325 in ZnT8 is highlighted in yellow. Residues involved in the charge interlock (Ch. Int.) are indicated in red text; notably, these residues are only partially conserved (Glu replacing Asp) between the bacterial and the vesicular ZnT subfamily (ZnT2, 3, 4 and 8). The CXXC motif, which is also specific to vesicular ZnTs, is highlighted in purple. Dileucine motifs in ZnT2 and 3 are purportedly involved in protein localisation 34. The ligands forming the purported third weaker zinc‐binding site in CzrB 17 are not indicated. (B) 3D homology model of human ZnT8cR based on *Thermus thermophilus* CzrB using swiss‐model (Materials and methods), highlighting the conserved metal‐binding ligands in magenta with bound zinc ions in grey and the T2D‐risk variant residue R325 in red. The triple β‐sheet face is predicted to form the dimer interface, while residue 325 is located in a loop at the apex of the dimer.

Based on a comparison of the primary sequences of several human and two bacterial CDF CTDs, for which 3D structures are known, the two ZnT8 CTD variants (ZnT8cR and ZnT8cW) are predicted to adopt an αββαβ fold (Fig. 1A) observed in at least four bacterial CTDs of homologous zinc transporters. This fold is characteristic of the ‘heavy metal‐associated domain’, also called the ferredoxin fold βαββαβ in different metalloproteins interacting with iron, copper or zinc 25. Indeed, we predict such a structure for all mammalian ZnT CTDs with the possible exception of ZnT9 (Fig. 1A). A 3D model of the ZnT8cR homodimer based on the structure of *T. thermophilus* CzrB was constructed (Fig. 1B). The model predicts that residue 325 is located in a loop which is in close proximity to the second protomer in the dimer in the zinc‐bound state.

Our analysis further shows that in the CTDs of human ZnTs, the ligands for a second metal ion in the binuclear site C are not strictly conserved and, importantly, the ligand stemming from the other subunit, His261 in *E. coli* YiiP, is not conserved (Fig. 1A). Therefore, an important issue is if the CTDs of these human transporters bind fewer or even no metal ions at all. The conservation of only three predicted metal ligands, that is, two histidines and one glutamate/aspartate in the vesicular ZnTs (Fig. 1A), raises the questions of whether the CTDs in these human transporters sense zinc ion concentrations and how the protomers forming the dimer interact. The metal ligands that are conserved do not form a bridge between the two protomer CTDs in the dimer; thus, the CTD dimerisation‐induced conformational change seen upon zinc binding to the CTD in *E. coli* YiiP 13 may not occur and may not have the same consequences in human ZnTs.

Remarkably, there is a high density of potential metal binding residues in the C‐terminal tail of ZnT8, including a CXXC motif, which is present only in the vesicular subfamily of human ZnTs (ZnT2, 3, 4 and 8). This motif is conserved in all verified vesicular ZnT sequences available from the UniProt database, including mouse, rat, cow and frog. The significance of this motif is not known although CXXC motifs have redox functions or a metal‐binding role in metalloproteins, such as in some copper chaperones where they can mediate metal transfer to client proteins 26. However, in copper chaperones, this motif is typically in a different position in the primary sequence.

A ‘charge interlock’ (Ch. Int.) comprised of Asp207 in the CTD and Lys77 in the TMD is thought to be important for dimer formation in the full‐length *E. coli* YiiP protein 13. However, these residues are not conserved in non‐vesicular human ZnTs (i.e. not ZnT2–4 or 8). The charge of these residues is conserved in vesicular ZnTs, but Asp207 in the *E. coli* YiiP CTD is replaced by Glu in the vesicular ZnT subfamily (Fig. 1A), while the TMD Lys77 is replaced by Arg.

### Protein yield

**Figure 2:**
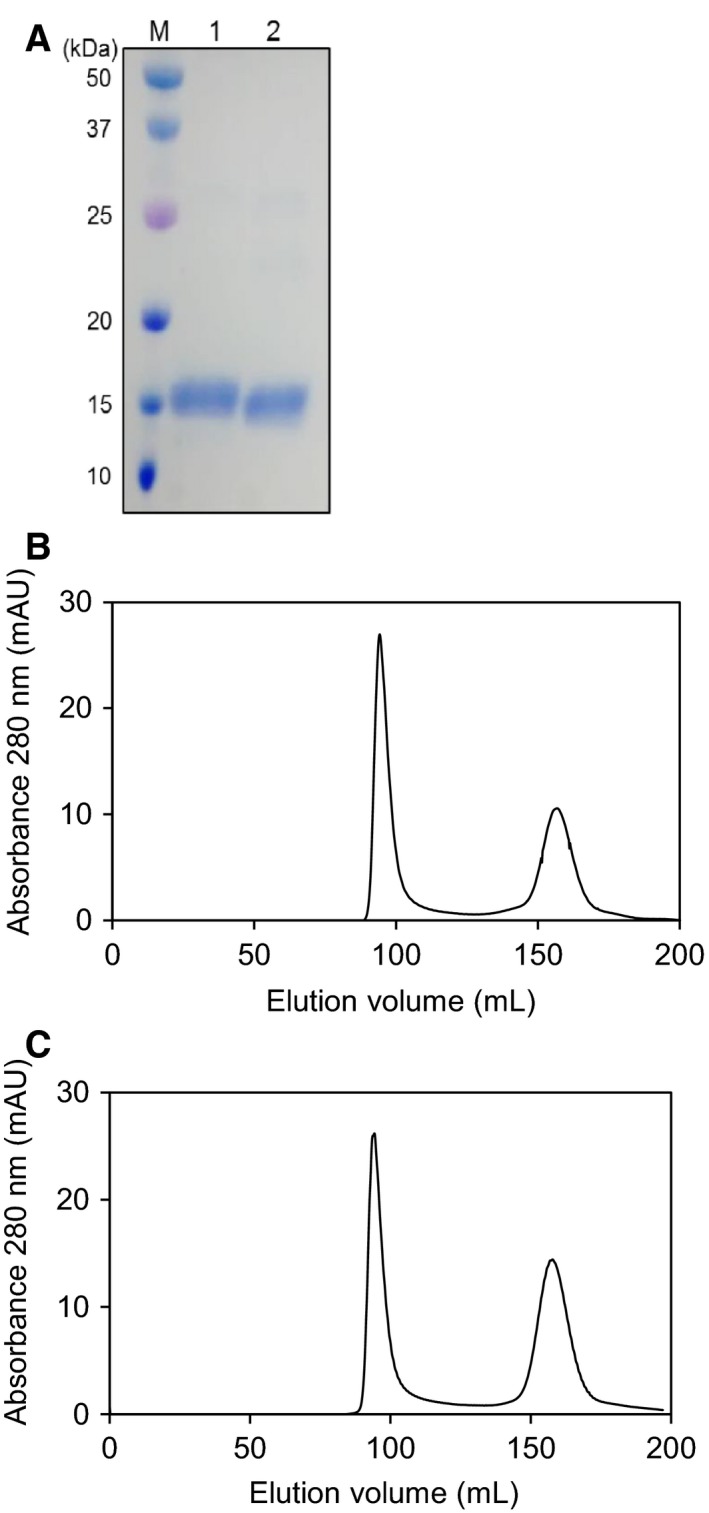
Purity and elution profiles of human ZnT8 CTD proteins. (A) Protein in the minor elution peaks at ~ 160 mL was analysed by SDS/PAGE and is > 95% pure ZnT8 CTD. Lane ‘M’ contains molecular weight markers; lane ‘1’ contains purified apo‐ZnT8cR; and lane ‘2’ contains purified apo‐ZnT8cW. The protein in the major elution peaks at ~ 95 mL was also analysed by SDS/PAGE (not shown) and is aggregated ZnT8. (B) Size exclusion chromatogram using a Superdex S75 26/60 column for ZnT8cR protein and, (C) ZnT8cW protein. Following calibration of the column (Materials and methods), the proteins in the fractions eluting at ~ 160 mL have a molecular mass of 34.9 kDa (calculated ZnT8 CTD monomer mass is 13.3 kDa).

A typical 2 L bacterial culture (of either variant, aa267–369 in addition to an N‐terminal hexahistidine tag and a TEV protease cleavage site) yielded ~ 1 mg of > 95% pure ZnT8 CTD protein (Fig. 2A). Protein samples were concentrated to ~ 100–300 μm. There is a tendency for the proteins to aggregate and ultimately precipitate completely after a period of ~ 2 weeks. To alleviate the aggregation issues, many buffer constituents and several different *E. coli* expression strains were screened; the most effective conditions for expression of a folded protein were used herein (Materials and methods). Addition of fresh Tris(2‐carboxyethyl)phosphine hydrochloride (TCEP) during the size exclusion purification step is necessary to obtain a high yield of pure protein.

### The two CTD variants share structural similarities

**Figure 3:**
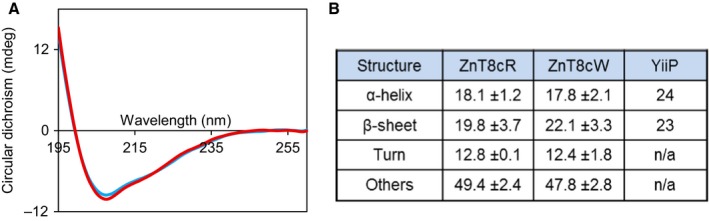
CD spectroscopy of the two human ZnT8 CTD variants. (A) Representative (*n* = 3) far‐UV CD spectra of 0.2 mg·mL^−1^ apo‐ZnT8cR(blue) and apo‐ZnT8cW (red) variants in 10 mm K_2_ HPO_4_, 60 mm NaCl, 20 mm sucrose, pH 8. Separate far‐UV CD spectra measured in the presence of two molar equivalents of Zn^2+^, two molar equivalents of Ni^2+^ or with KCl replacing NaCl, were not significantly different from those of the apo‐proteins. (B) Protein secondary structure, expressed as % ± standard deviation (*n* = 3), was determined using individual CD spectra for both apo‐ZnT8cR and ZnT8cW variants with the BeStSel algorithm (Materials and methods). The difference in secondary structure between the two variants is not statistically significant. Helix and sheet content of *Escherichia coli* YiiP CTD were calculated from the 3D structure (PDB ID: 2qfi), while turns and other structures could not be readily differentiated.

ZnT8cR and ZnT8cW elute at the same volume in size exclusion chromatography (160 mL, Fig. 2B,C). Calibration of the Superdex S75 26/60 column with protein standards (Materials and methods) indicates that both variant ZnT8 CTD proteins have an apparent molecular mass of 34.9 kDa. The expected mass of the monomer is 13.3 kDa including the His‐tag and TEV protease site. Native PAGE analyses of the purified proteins indicate that both variants are dimeric. SDS/PAGE analysis of the largest peak at 95 mL indicates that it is aggregated but soluble ZnT8 CTD protein. The secondary structure of both apo‐ZnT8 CTD variants was investigated using CD spectroscopy; the two variants yield similar far‐UV CD spectra (Fig. 3A). The spectra did not change significantly upon addition of two molar equivalents of ZnCl_2_ or NiSO_4_, or replacement of NaCl with KCl. Inserting individual CD spectra into BeStSel 27, a fold recognition algorithm, showed that the two variants contain similar helix and sheet content to both each other and the CTD of the 3D‐characterised *E. coli* homologue YiiP (Fig. 3B). Thus, as predicted, the secondary structure and fold are highly conserved.

### ZnT8cR is more thermostable than ZnT8cW; Zn^2+^ stabilises both variants

**Figure 4:**
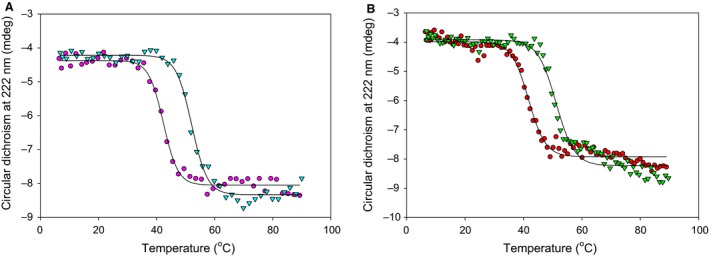
Thermostability of the two human ZnT8 CTD variants. (A) Representative (*n* ≥ 3) melting curves for apo‐ZnT8cR (magenta circles, *T*_m_ = 42.8 ± 0.5 °C) and ZnT8cR with two molar equivalents of Zn^2+^ (teal triangles, *T*_m_ = 54.5 ± 2.1 °C) measuring the change in CD at 222 nm from 6 to 92 °C with a heating rate of 1 °C·min^−1^. (B) Representative (*n* = 3) CD melting curves of apo‐ZnT8cW (red circles, *T*_m_ = 41.4 ± 0.4 °C) and ZnT8cW in the presence of two molar equivalents of Zn^2+^ (green triangles, *T*_m_ = 51.0 ± 1.8 °C). There are significant differences between thermal stability of apo‐ZnT8cR and apo‐ZnT8cW (*n* = 3, *P* = 0.013) and between both apo‐variants and the variant in the presence of Zn^2+^ (for each comparison *n* = 3, *P* < 0.001). The difference in stability between the two variants in the presence of Zn^2+^ is not statistically significant (*P* = 0.093).

The thermal stability of both CTD variants in the presence and absence of ZnCl_2_ was investigated using melting analysis by both CD spectroscopy between 6 and 92 °C (Fig. 4A,B) and nano differential scanning fluorimetry (nDSF) between 20 and 85 °C. This type of DSF utilises intrinsic protein fluorescence; the ratio of the emission at 350 nm to that at 330 nm as a function of temperature reveals the point(s) at which the protein structure changes significantly. There is a significant difference between the stability of the CTD variants in the apo (Zn^2+^‐free) form as measured with both CD and nDSF; the ZnT8cR *T*_m_ is 42.8 ± 0.5 °C, whereas the ZnT8cW *T*_m_ is 41.4 ± 0.4 °C (*n* = 3, *P* = 0.013). Remarkably, apo‐ZnT8cR (T2D‐risk in the full‐length protein) has higher thermostability than apo‐ZnT8cW (T2D‐protective in the full‐length protein). Both CTD variants are significantly more stable in the presence of two molar equivalents of Zn^2+^; ZnT8cR‐2Zn *T*_m_ is 54.5 ± 2.1 °C and ZnT8cW‐2Zn *T*_m_ is 51.0 ± 1.8 °C (in each comparison *n* = 3, *P* < 0.001), but not in the presence of two molar equivalents of Ni^2+^. The numerical difference in stability between the two CTD variants in the presence of Zn^2+^ is not statistically significant (*P* = 0.093).

### The two Trp residues in ZnT8cW are in different local environments

**Figure 5:**
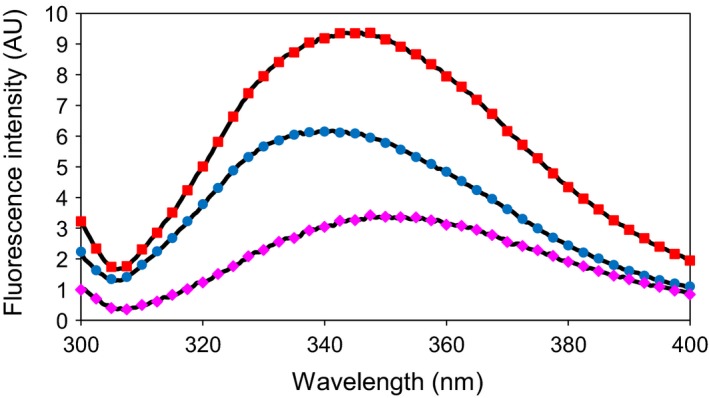
Fluorescence spectroscopy of the two human ZnT8 CTD variants. Representative (*n* = 3) fluorescence spectra of ZnT8cW (red squares) and ZnT8cR (blue circles) protein, both 2.8 μm, in 50 mm Tris/HCl, pH 8, 300 mm NaCl (λ_Ex_ = 295 nm). The ZnT8cR variant contains one tryptophan residue (W306), while the ZnT8cW variant contains two (W306 and W325). Therefore, by subtracting the ZnT8cR signal from that of ZnT8cW the fluorescence spectrum of W325 was obtained (magenta diamonds).

ZnT8cW contains two tryptophan residues (W306 and W325), whereas ZnT8cR contains only one (W306). The emission spectrum (λ_Ex_ = 295 nm) of ZnT8cR provides information on the tryptophan residue shared by both variants (i.e. W306). Therefore, by subtracting the ZnT8cR emission spectrum from that of ZnT8cW, information about W325 in ZnT8cW can be obtained (Fig. 5). The emission maximum of ZnT8cR was 340 nm, corresponding to W306, while that of ZnT8cW was 345 nm. The emission maximum of W325, calculated by subtracting the ZnT8cR spectrum from that of ZnT8cW, is 350 nm. For comparison, a pure *N*‐acetyl‐dl‐tryptophan solution measured in the same buffer has an emission maximum at 363 nm. The degree of blue shift of a tryptophan residue's emission from that of pure tryptophan in solution depends on how hydrophobic the local environment is. In the ZnT8cW protein, W325 is therefore in a less hydrophobic environment than W306, and therefore more solvent accessible.

### The amino acid at position 325 affects dimer formation

**Figure 6:**
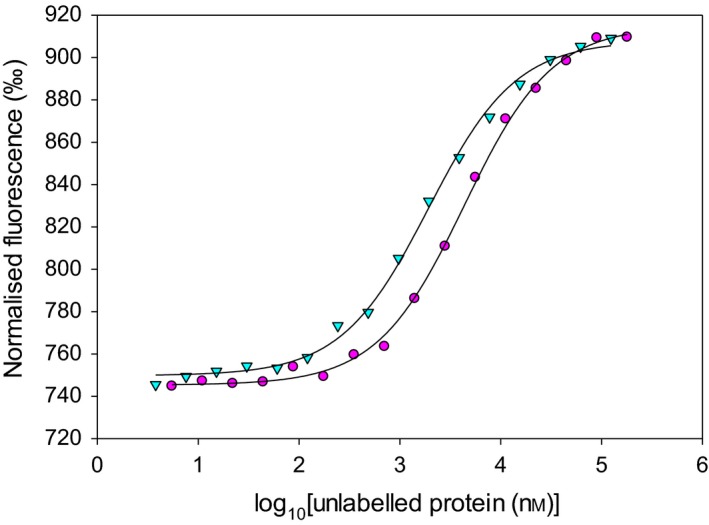
Dimerisation of the two human ZnT8 CTD variants. Representative (*n* = 3) MST traces for dimerisation of ZnT8 CTD protein. Fluorescently labelled apo‐ZnT8cR (100 nm, magenta circles) was titrated (in the presence of 1 mm EDTA) with unlabelled apo‐ZnT8cR protein (180 μm–5.5 nm), yielding a homodimerisation *K*_d_ of 4.3 ± 1.3 μm. Fluorescently labelled apo‐ZnT8cW (100 nm, teal triangles) was titrated (in the presence of 1 mm EDTA) with unlabelled apo‐ZnT8cW protein (124 μm–3.8 nm), with a homodimerisation *K*_d_ of 1.8 ± 0.1 μm. There is a significant difference between the homodimerisation *K*_d_ of each variant in the presence of EDTA (*n* = 3, *P* = 0.034).

The homodimerisation affinities of both ZnT8 CTD variants were measured using microscale thermophoresis (MST) in the presence of EDTA, eliminating any influence of divalent metal ions (Fig. 6). Titrating 100 nm labelled apo‐protein with 180 μm–5.5 nm (ZnT8cR) or 124 μm–3.8 nm (ZnT8cW) unlabelled apo‐protein yielded homodimerisation *K*_d_ values of 4.3 ± 1.3 μm for ZnT8cR and 1.8 ± 0.1 μm for ZnT8cW. This difference is statistically significant (*n* = 3, *P* = 0.034). Thus, the dimerisation of ZnT8cR (T2D‐risk in the full‐length protein) occurs with less affinity than ZnT8cW (T2D‐protective in the full‐length protein) in the presence of EDTA. The directionality of this difference is opposite to that observed for the thermostability of the two forms.

### The amino acid at position 325 does not directly affect metal binding

**Figure 7:**
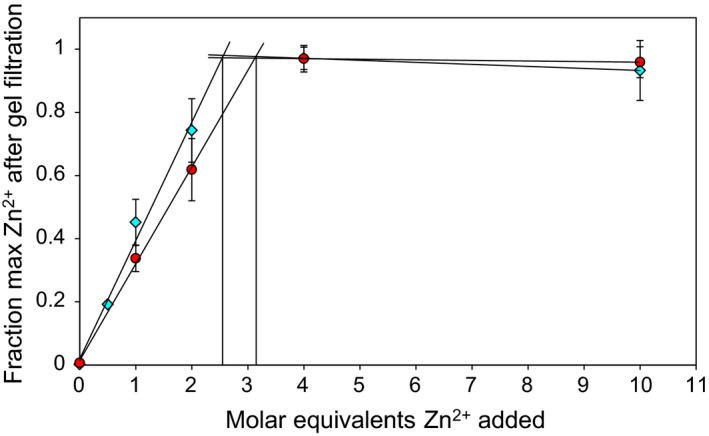
Zinc stoichiometry of the two ZnT8 CTD variants. Fraction of the maximum Zn^2+^ content of 10 μm ZnT8cR (teal diamonds) and ZnT8cW (red circles) following incubation with 0–10 molar equivalents of Zn^2+^ and subsequent gel filtration to remove unbound Zn^2+^. Protein concentration was determined spectrophotometrically (Materials and methods). The intersection points in the titration data indicate that ZnT8cR binds Zn^2+^ with a stoichiometry of 2.6 ± 0.4 per monomer, whereas ZnT8cW binds 3.2 ± 0.5 per monomer. The difference between the two variants is not statistically significant (*n* = 3 for both variants, *P* = 0.156).

Based on sequence analysis, the expected divalent metal ion binding capacity for ZnT8 CTDs is one ion per monomer (Fig. 1A). The two variant apo‐proteins (10 μm protein) were incubated with 0–10 molar equivalents of Zn^2+^ and subjected to gel filtration to remove any loosely bound zinc. Inductively coupled plasma mass spectrometry (ICP‐MS) analysis of the apo‐ZnT8 CTD proteins incubated with no additional Zn^2+^ showed that 0.21 ± 0.07 (*n* = 6) divalent metal ions (Zn^2+^ and Ni^2+^) were residually bound per monomer. The vast majority of this residual metal load (> 90%) was contributed by Ni^2+^. Supplementing up to 10 molar equivalents of Zn^2+^ indicates that both variants bind approximately three Zn^2+^ ions per monomer; an average of 2.6 ± 0.4 Zn^2+^ ions bind to ZnT8cR, whereas 3.2 ± 0.5 Zn^2+^ ions bind to ZnT8cW (Fig. 7). This difference between the two variants is not statistically significant (*n* = 3, *P* = 0.156). Upon addition of 4–10 molar equivalents of Zn^2+^, the small amount of Ni^2+^ residually bound to both CTD variants was displaced.

**Figure 8:**
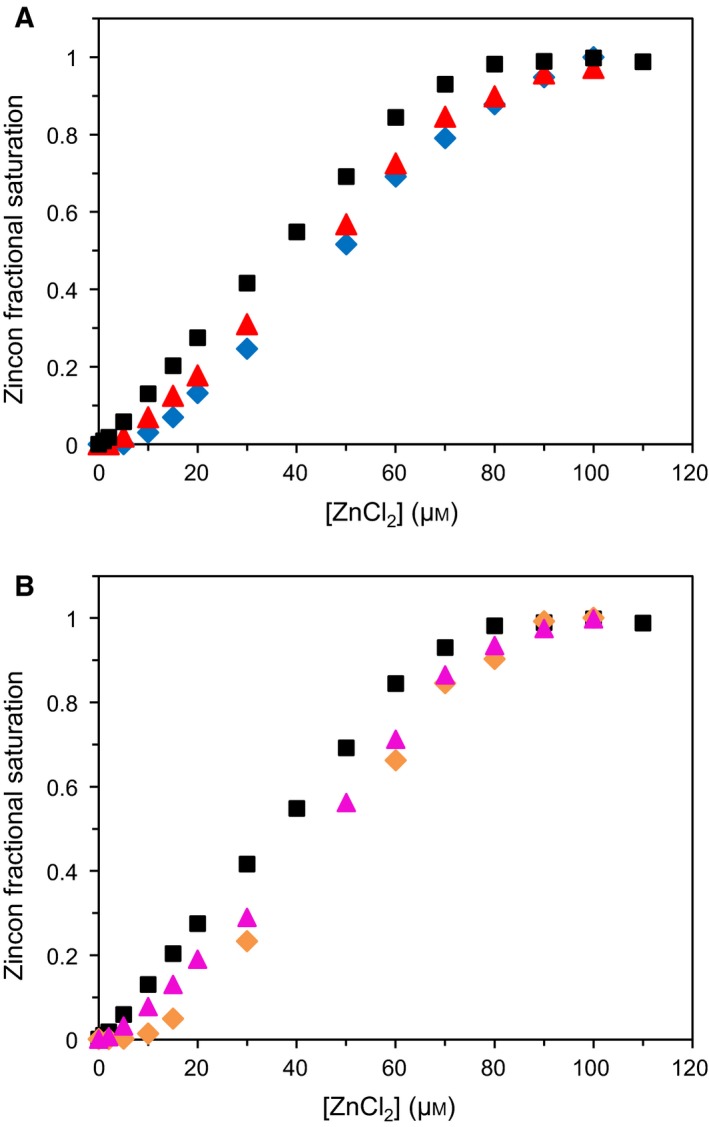
Zinc affinity of the two ZnT8 CTD variants. (A) Zinc binding to ZnT8cR in competition with Zincon. Measuring absorbance of 620 nm, it takes 70 μm ZnCl_2_ to saturate 70 μm Zincon in 50 mm HEPES, pH 8, 300 mm NaCl, 100 mm sucrose (black squares). In competition with 5 μm apo‐ZnT8cR (blue diamonds), no signal at 620 nm is detected until 10 μm ZnCl_2_ is added, revealing two high affinity zinc‐binding sites in ZnT8cR which outcompete Zincon. An additional 75 μm ZnCl_2_ is required to fully saturate Zincon, identifying one lower affinity (~ μm) site that directly competes with Zincon. When ZnT8cR is reduced and incubated with iodoacetamide for 1 h prior to the Zincon assay (red triangles), only 5 μm ZnCl_2_ is required to elicit the initial signal at 620 nm. An additional 75 μm ZnCl_2_ is required to saturate the Zincon. Thus, when the cysteines are blocked by alkylation, ZnT8cR retains one high affinity and one low affinity Zn^2+^‐binding site. B, Zinc binding to ZnT8cW in competition with Zincon. ZnCl_2_ titration of Zincon alone in HEPES buffer (black squares), in competition with apo‐ZnT8cW (orange diamonds), and in competition with ZnT8cW modified with iodoacetamide (magenta triangles), demonstrating that ZnT8cW also contains two high affinity and one low affinity zinc binding sites, and that one high affinity binding site is lost upon alkylation of the three cysteines in ZnT8cW.

A competition assay with the chromophoric chelating agent Zincon shows similar results for both ZnT8 CTD variants (Fig. 8A,B). When titrated with zinc in buffer alone, 70 μm Zincon is saturated with 70 μm ZnCl_2_ and an initial increase in absorbance at 620 nm is measured upon addition of 1 μm ZnCl_2_. Zincon has a *K*_d_ of 214 nm for a 1 : 1 complex with zinc at pH 8 28. However, when competing with 5 μm apo‐ZnT8 CTD (either variant), the initial increase in absorbance is not seen until 10 μm Zn^2+^ is added, indicating that both ZnT8 CTD variants contain two Zn^2+^‐binding sites that have a tighter affinity than 214 nm and thus outcompete the zinc binding to Zincon. Following this initial 10 μm ZnCl_2_, an additional 75 μm ZnCl_2_ is required to saturate zinc binding to Zincon in the presence of 5 μm apo‐ZnT8 CTD protein (both variants). Thus, both variants have an additional zinc site with low affinity competing directly with Zincon. When both ZnT8 CTD protein variants have their cysteines blocked by alkylation with iodoacetamide, only 5 μm ZnCl_2_ is required to measure a change in absorbance at 620 nm. This result indicates that cysteines in the C‐terminal tail, which contains three cysteines, constitute one of the two high affinity binding sites that outcompete the binding of zinc to Zincon. With protein modified by iodoacetamide (both variants), an additional 75 μm ZnCl_2_ is still required to saturate the Zincon, indicating that the lower affinity site is not lost upon cysteine alkylation.

### A dityrosine bond does not form between ZnT8 CTD protomers

Using a specific anti‐dityrosine antibody, an inter‐protomer dityrosine bond in the CTDs of ZnT3 and ZnT4 homodimers was detected 29. Dityrosine bonds have a high quantum yield at 407 nm when using an excitation wavelength of 325 nm, well above the excitation maximum of individual tyrosine residues. There is one tyrosine residue in ZnT8 CTD (Y284) although it is not at the same position as the three tyrosine residues involved in ZnT3 and ZnT4 homodimerisation. Nevertheless, using fluorescence spectroscopy, we could not detect any emission associated with a dityrosine in either ZnT8 CTD variant.

## Discussion

The mammalian ZnTs are thought to function with the Zn^2+^/H^+^ antiport mechanism elucidated for ZnT1 and the bacterial homologues 30. The antiport is likely coupled to induced conformational changes that alternately open the channel inward or outward as shown for the bacterial homologues 13, 16. In contrast to the *E. coli* YiiP protein, which has a zinc/cadmium selectivity filter in the TMD with one histidine and three aspartates, the mammalian ZnTs utilise two histidine and two aspartate side chains to transport zinc specifically 31. Amongst mammalian ZnTs (with the exception of ZnT10, which has an asparagine instead of one of the two aspartates in the TMD and accordingly transports manganese in addition to zinc 32), the zinc transport site and the overall structure of the TMD are highly conserved 3. The CTD, however, is much more variable and is thought to be critical in the evolution of these transporters for different functions, such as the subset of four vesicular transporters, ZnT2–4 and ZnT8. This subgroup supplies exocytotic vesicles with zinc for various purposes, such as synaptic vesicles (ZnT3) involved in neurotransmission 33 and vesicles in mammary epithelial cells (ZnT2) that supply zinc in the milk of lactating women 34. How ZnTs acquire and deliver sufficient zinc to exocytotic vesicles is an unresolved biochemical issue.

Despite the lack of high sequence homology between CTDs in mammalian ZnTs, various structural features are conserved, for instance the overall fold. Based on prediction of secondary structure and CD data, both ZnT8cR and ZnT8cW form the αββαβ structure observed in the structure of *E. coli* YiiP, and most other ZnT CTDs are predicted to adopt this structure (Fig. 1A). Referred to as a ‘ferredoxin’ fold as it was originally found in iron proteins, it is also commonly found in copper proteins, in particular copper chaperones 25. However, the metal‐binding sites are at different positions in these proteins.

Due to the predicted location of ZnT8 residue 325 at the CTD dimer interface (Fig. 1B), the R325W replacement is likely to affect dimer formation and stability. A significant difference in dimer association between the two human ZnT8 CTD variants was detected in this investigation. The directionality of the difference was not expected, however; despite an increased thermostability of ZnT8cR, its dimerisation affinity was lower than that of ZnT8cW. Collectively, these data show that both dimer formation and stability are affected in the two CTD variants. The 2.9‐Å crystal structure of *E. coli* YiiP revealed that the CTD dimer interface is highly charged and that in the absence of bound Zn^2+^_,_ the repulsive charges in the dimer cause the protomers to swing apart 13. The exchange of the charged R325 residue for uncharged W325 may disrupt this charge interaction in ZnT8, and may explain the biophysical differences in the two variant CTDs observed. Neither the Arg nor the Trp at position 325 are conserved among the ZnTs, or even among species; murine and rat *ZnT8* encode Gln325. The position is at a variable loop between the conserved secondary structures.

The identity of residue 325 in ZnT8 alters the specificity of ZnT8 autoantibodies in T1D 24, with sera from at least 22% of patients reacting with one of either R325 or W325 antibodies but not the other. Previous studies expressing ZnT8 CTDs to investigate antibody epitopes did not prove that the protein folds correctly 35. A properly folded protein may be crucial for presenting the correct 3D epitope to the antibody. Indeed, the ZnT8 autoantibody epitope has been confirmed to be conformational rather than linear 24. Therefore, the availability and characterisation of the folded CTDs may be important for efficient antibody production.

Another model for the dimerisation in mammalian vesicular ZnTs, namely the formation of a dityrosine, has been advanced for ZnT3 29. The ZnT8 CTD contains one tyrosine (Y284) although its location in the primary sequence is not conserved with any of the tyrosine residues implicated in ZnT3 homodimerisation. We found no evidence for dityrosine bond formation in either ZnT8 CTD variant. A charge interlock with residues from both the TMD and CTD serves as a hinge in the dimerisation of full‐length CDF proteins 13. The charge interlock CTD residues (albeit Glu replacing Asp207 and Arg replacing Lys77 in YiiP) are conserved in vesicular ZnTs (Fig. 1A) but, because of the absence of the TMD, isolated CDF CTDs do not interrogate this aspect of intersubunit linkages. Intriguingly, these charge interlock residues are not conserved in non‐vesicular ZnTs, suggesting that the intersubunit linkages differ amongst mammalian ZnTs.

A characteristic feature of CTDs in bacterial CDFs is two zinc‐binding sites per monomer, harbouring four zinc ions in the dimer 12 (although the *T. thermophilus* CzrB CTD contains an additional weak zinc‐binding site 17). One of these sites utilises ligands from both protomers, therefore bridging between the dimer subunits, while the other(s) are formed of ligands from only one protomer. Both metal‐binding sites utilise a water molecule as the fourth ligand in the tetrahedral coordination of the Zn^2+^ ions. Remarkably, the ligands for the intersubunit metal‐binding site are not conserved in the human ZnTs (Fig. 1A). Specifically, a ligand corresponding to His261 is missing. This is the only residue contributing a metal‐binding ligand from the second protomer in the dimer in *E. coli* YiiP, and is involved in the CTD conformational changes seen upon zinc binding, or ‘zinc sensing’, when the cytosolic zinc concentration reaches an upper threshold 13. The primary biological function of these bacterial transporters is to protect the cytosol from zinc overload, and current evidence suggests micromolar *K*_m_ values for transport 13. The problem with this model for the four vesicular ZnTs (ZnT2–4 and 8) is that there is only picomolar free zinc available in the cytosol of human cells, and the total vesicular zinc concentrations are high millimolar. Therefore, either the vesicular ZnT CTDs are able to sense much lower cytosolic zinc concentrations than their bacterial homologues, for which there is no evidence at present, or the role of the CTD is different from that of the bacterial proteins and not involved in sensing zinc directly, as suggested by our findings.

Our measurements show that both apo‐ZnT8 CTD variants form stable dimers. Addition of two molar equivalents of zinc significantly increases the stability of both variant CTDs, without significantly altering their secondary structures. Following zinc addition up to saturation with 10 molar equivalents of zinc, three zinc ions were tightly bound per protein monomer. The difficulty in relating the metal binding to a particular binding site in the CTD stems from the fact that the expressed protein has a hexahistidine tag. It was possible to remove this tag, but the resulting protein was unstable and precipitated, rendering further experimentation impossible. ZnT8 has three C‐terminal cysteine residues, including a CXXC motif that has been shown to bind zinc in the metal‐binding domains of *E. coli* ZnTA 36 and *Synechocystis* PCC 6803 ZiaA 37, and copper in copper chaperones 38. Amongst human ZnTs, the CXXC motif is only conserved in the vesicular subfamily (Fig. 1A). Competition assays performed with the chromophoric zinc chelator Zincon and protein modified with iodoacetamide (Fig. 8) reveal that one of the two < 214 nm affinity zinc‐binding sites identified in both ZnT8 CTD variants is formed of the C‐terminal cysteines. The small amount of residual Ni^2+^ that was bound to both variant apo‐proteins was only displaced upon supplementing the protein with 4–10 molar equivalents of zinc. This agrees with published data indicating that the His‐tag has a higher affinity for Ni^2+^ than it does for Zn^2+^ 39. Protein‐bound His‐tags bind Ni^2+^ with an affinity of ~ 700 nm 40. These data support the hypothesis that the low affinity site (approximately micromolar), identified in both ZnT8 CTD variants with the Zincon competition assay, is contributed by the His‐tag. Therefore, our metal‐binding data can be reconciled with the prediction from the sequence alignment that indeed maximally only one metal ion binds with high affinity at the canonical interface site in the protein as isolated, the second with high affinity at the C‐terminal cysteines and the third with lower affinity to the His‐tag.

A recent report, in which the activity of ZnT8 reconstituted in liposomes was investigated, concluded that the transport activity is dependent on the lipid environment, and inferred that the lipid environment affects zinc loading during insulin granule biogenesis 9. This report also noted that the T2D‐risk R325 ZnT8 variant consistently showed a small increase in zinc transport activity compared to the T2D‐protective W325 variant, which was revealed only with specific lipid compositions of the liposomes. In accordance with higher transport activity, it was noted that human islets with the R325 ZnT8 variant had a higher zinc content 41. Another report on ZnT8 transport in *Xenopus laevis* oocytes did not detect a difference in transport kinetics between the ZnT8 R/W325 variants, supporting the conclusion that the liposome lipid composition may be crucial for revealing differences between the two variants 42.

There are two main conclusions. First, the mammalian vesicular ZnTs are significantly different from bacterial CDF ZnTs in their CTD zinc binding. The loss of the subunit‐bridging ‘sensing’ zinc binding site in the CTD, the additional high affinity zinc binding at the C‐terminal cysteines and the disparity between the very low concentration (pm) of free cytosolic zinc and the very high (mm) total zinc levels found in secretory vesicles, strongly suggest that the sensing of excess cytosolic zinc and the concomitant transport in bacteria would need to function differently in mammalian systems supplying zinc to exocytotic vesicles. Bacterial zinc exporters need only function when the cell is experiencing high and/or toxic levels of zinc, whereas loading of insulin and other secretory vesicles, for instance synaptic vesicles by ZnT3 33, must occur under conditions of normal cytosolic zinc concentrations. Second, this is the first report detailing that the W325R mutation causes significant differences in ZnT8 CTD dimer formation and stability. How or even if these effects in the CTDs lead to the altered transport function of the full‐length proteins reported elsewhere 9, and ultimately to the relatively large increased risk of developing T2D for carriers of the R325 variant, will require further investigation. That the T2D‐risk ZnT8 R325 variant is the more active form of the transporter suggests that people with the R325 variant may have an increased zinc content in their insulin granules as indicated by the data on human islets 41. This increased granular zinc uptake may deplete cytosolic zinc and affect β‐cell function. If so, it may need to be ameliorated with a higher dietary zinc intake 43.

## Materials and methods

### Materials

Tris(2‐carboxyethyl)phosphine hydrochloride, HEPES, iodoacetamide, Zincon sodium salt, NaCl, K_2_HPO_4_, KH_2_PO_4_, MgCl_2_, ZnCl_2_ and *N*‐acetyl‐dl‐tryptophan were purchased from Sigma Aldrich (St. Louis, MO, USA); Tris‐base and SDS from Severn Biotech (Kidderminster, UK); DTT and PMSF from Thermo Fisher Scientific (Waltham, MA, USA); Tween‐20 and NiSO_4_.6H_2_O from Acros Organics (Geel, Belgium); sucrose from Merck Millipore (Burlington, MA, USA); IPTG from Promega (Madison, WI, USA); l,l‐dityrosine dihydrochloride from Santa Cruz Biotechnology (Dallas, TX, USA); 5,5′‐dithiobis(2‐nitrobenzoic acid) (DTNB; Ellman's reagent) from Invitrogen (Carlsbad, CA, USA); EDTA from Cambridge Bioscience (Cambridge, UK); LB media powder from MP Biomedicals (Santa Ana, CA, USA); and imidazole from Apollo Scientific (Stockport, UK).

### Protein expression and purification

The sequence encoding residues 267–369 of human R325 ZnT8 (ZnT8cR; any residue numbering refers to full‐length human ZnT8 long isoform, Ensembl transcript *SLC30A8‐002*) was optimised for *E. coli* expression and the cDNA synthesised by DNA2.0 (Menlo Park, CA, USA). It was inserted into pET6H encoding an N‐terminal hexahistidine tag and a TEV protease cleavage site. Mutagenesis to produce the W325 variant (ZnT8cW) was carried out by Mutagenex (Suwanee, GA, USA) using PCR‐based substitution, followed by sequence verification of the inserts of both plasmids. The two plasmids were transformed into *E. coli* strain SoluBL21™ (AMS Biotechnology, Abingdon, UK) and grown at 30 °C in LB media containing 100 μg·mL^−1^ ampicillin until the OD_600_ reached 0.60. Cells were then kept at 16 °C on an orbital shaker (G25 Incubator Shaker, New Brunswick, Edison, NJ, USA; 210 r.p.m.) for 30 min before protein expression was induced with 0.5 mm IPTG and the cells kept at 16 °C and at 210 r.p.m. for an additional 42 h. Cells were harvested by centrifugation and resuspended in 10 mL lysis buffer [50 mm Tris/HCl, pH 8, 100 mm NaCl, 100 mm sucrose, 5 mm DTT, 2 mm MgCl_2_, 1 mm PMSF, 5 U·mL^−1^ DNase (Thermo Fisher Scientific)] until a homogenous solution was obtained. The homogenate was diluted 1 : 6 with equilibration buffer [50 mm Tris/HCl, pH 8, 100 mm NaCl, 100 mm sucrose, 2 mm DTT, 20 mm imidazole, containing one tablet of Complete ULTRA mini EDTA‐free protease inhibitors (Roche, Basel, Switzerland)], and sonicated (Model 2000U, Ultrasonic Power Corp. (Freeport, IL, USA); +285 output, 0.5 s^−1^ pulse) in an ice‐water bath for 20 s pulse and 40 s rest settings for a total of 15 min, followed by centrifugation at 45 000 ***g*** for 40 min at 4 °C. The 60 mL supernatant was added to 2 mL of pre‐equilibrated Ni^2+^ affinity gel (Sigma Aldrich) and incubated end‐over‐end on a roller at 4 °C for 40 min. After centrifugation at 500 ***g*** for 1 min at 4 °C, the pellet was washed three times with equilibration buffer containing 500 mm NaCl. The bound protein was eluted in five 1 mL washes with elution buffer (50 mm Tris/HCl, pH 8, 500 mm NaCl, 100 mm sucrose, 2 mm DTT, 300 mm imidazole). The eluate was loaded onto a Superdex S75 26/60 column (GE Healthcare, Chicago, IL, USA) equilibrated with purification buffer (50 mm Tris/HCl, pH 8, 300 mm NaCl, 100 mm sucrose, 100 μm TCEP) using a flow of 2.2 mL·min^−1^ and a column temperature of 4 °C. The column was calibrated with yeast alcohol dehydrogenase, bovine serum albumin, bovine erythrocyte carbonic anhydrase and aprotinin. To produce ZnT8c apo‐protein, 2 mm TCEP and 1 mm EDTA were added to the 10 mL fractions from size exclusion chromatography and then concentrated to ~ 0.5 mL using a 15 mL 3 kDa MWCO centrifugal concentrator (Merck Millipore). Samples were stored in this state for up to 1 week at 4 °C. Up to 0.5 mL protein was then diluted 30× with EDTA‐ and TCEP‐free buffer (exact constituents dependent on assay to be carried out as given in the corresponding experiments) and concentrated to 0.5 mL with the same centrifugal concentrators, performing this dilution/concentration step at least three times. Reduced apo (zinc‐free) protein was then used fresh within 1 day. Concentrated samples were analysed using 16% (w/v) Tris‐glycine SDS/PAGE (Thermo Fisher Scientific) 44. Protein samples used for MST were purified as above, except that an amine‐free buffer (50 mm potassium phosphate, pH 8, 300 mm NaCl, 100 mm sucrose, 100 μm TCEP) was used for the size exclusion chromatography.

### Determination of protein concentration

The concentration of the protein samples was determined spectrophotometrically using a modified Bradford assay 45 with Bio‐Rad protein assay reagent (Hercules, CA, USA). Bovine serum albumin (Thermo Fisher Scientific) dissolved in purification buffer was used as the standard. The accuracy of the determination was corroborated by spectroscopic measurements, using extinction coefficients of 13 980 m ^−1^·cm^−1^ for ZnT8cW and 8480 m ^−1^·cm^−1^ for ZnT8cR (calculated from the primary protein sequence).

### CD spectroscopy

CD spectra were acquired on a Chirascan Plus spectrometer (Applied Photophysics, Leatherhead, UK). Far‐UV spectra in the 195–260 nm range were recorded using a 0.5 mm path length cuvette, a bandwidth of 2 nm, a step size of 1 nm and a time‐per‐point of 1 s. Spectra were taken at 20 °C. For all spectra, the protein concentrations were 0.2 mg·mL^−1^ in 10 mm potassium phosphate, pH 8, 60 mm NaCl, 20 mm sucrose. Protein secondary structure content based on CD spectra was determined using BeStSel 27. Melting analyses involved monitoring the CD signal at 222 nm between 6 and 92 °C at a heating rate of 1 °C·min^−1^. Separate far‐UV spectra and melting analyses were performed with both variants in the presence of two molar equivalents of ZnCl_2_, two molar equivalents of NiSO_4_ and by replacing the 60 mm NaCl with 60 mm KCl to examine any possible influence of the cation.

### Nano differential scanning fluorimetry (nDSF)

Samples of 5 μm ZnT8cR and ZnT8cW in 10 mm Tris/HCl, pH 8, 60 mm NaCl, 20 mm sucrose, 2 mm DTT were loaded into nDSF‐grade standard capillaries (NanoTemper Technologies, Munich, Germany). Protein unfolding was measured by detecting fluorescence emission changes at 350 and 330 nm, between 20 and 85 °C at a rate of 1 °C·min^−1^, using a Prometheus NT.48 instrument (NanoTemper Technologies). Protein melting temperatures were calculated by taking the first derivative of the 350/330 nm ratio, and are given as the average of at least three samples ± standard deviation.

### Microscale thermophoresis (MST)

Samples of 20 μm ZnT8cR or ZnT8cW were labelled with amine‐reactive Monolith NT.115 labelling kit Red‐NHS (#MO‐L001; NanoTemper Technologies) as per the manufacturer's instructions. Labelled proteins were diluted to a final concentration of 100 nm in 10 mm potassium phosphate, pH 8, 60 mm NaCl, 20 mm sucrose, 100 μm TCEP, 1 mm EDTA, 0.05% (v/v) Tween‐20. ZnT8cR‐Red and ZnT8cW‐Red were titrated with 180 μm–5.5 nm unlabelled ZnT8cR or 124 μm–3.8 nm unlabelled ZnT8cW, respectively. All experiments were performed using a Monolith NT.115 instrument (NanoTemper Technologies) at 25 °C in standard‐coated capillaries (NanoTemper Technologies). Binding affinities were calculated using MO.Affinity Analysis software (NanoTemper Technologies) and are expressed as the average of at least three separate measurements ± standard deviation.

### Inductively coupled plasma mass spectrometry (ICP‐MS)

Concentrated apo‐protein samples of both variants were prepared and diluted with ≥ 18.2 MΩ.cm water (ELGA LabWater, High Wycombe, UK). These samples were analysed for zinc and nickel with ICP‐MS (Perkin Elmer Life Science, Waltham, MA, USA, model NexION 350D) and were shown to have reduced metal content compared with protein samples that had not been incubated with additional TCEP and EDTA.

To measure zinc‐binding stoichiometry, ZnT8cR and ZnT8cW samples were prepared in 10 mm Tris/HCl, pH 8, 60 mm NaCl and 20 mm sucrose. Samples of each isoform were diluted to 10 μm. Each protein sample was then incubated with 0, 1, 2, 4 or 10 molar equivalents of Zn^2+^ (ZnCl_2_) for 10 min at 21 °C, followed by gravity‐flow gel filtration using PD MiniTrap G‐25 desalting columns (GE Healthcare) to remove excess zinc. Samples were then diluted with ≥ 18.2 MΩ.cm water and analysed for zinc and nickel using ICP‐MS.

### Fluorescence

Fluorescence measurements were taken at 21 °C using a Perkin Elmer LS50 fluorimeter. Separate ZnT8cR and ZnT8cW samples were diluted to 2.8 μm in 50 mm Tris/HCl, pH 8, 300 mm NaCl in a volume of 2 mL. Emission spectra (λ_Ex_ = 295 nm) were collected between 300 and 400 nm at a 100 nm·min^−1^ scan rate. Emission spectra for 3 μm *N*‐acetyl‐dl‐tryptophan solutions were collected in the same buffer. Emission spectra were recorded between 300 and 450 nm (λ_Ex_ = 325 nm) for examining the presence of a dityrosine bond, using 1 μm l,l‐dityrosine dihydrochloride in 1 m Tris/HCl, pH 8 as a positive control.

### Assessing redox state of the cysteines and alkylation of cysteine residues

DTNB (Ellman's reagent) was used to quantify the free sulfhydryls present in the protein samples, following the manufacturer's instructions, using 50 mm HEPES, pH 8, 300 mm NaCl and 100 mm sucrose as the reaction buffer and the molar extinction coefficient at 412 nm of TNB (14 150 m ^−1^·cm^−1^). Protein was diluted to 2 μm and the production of TNB measured after 15 min. Iodoacetamide was used to alkylate the sulfhydryls on the cysteine side chains, as per the manufacturer's instructions. Alkylation was confirmed upon measuring no free sulfhydryls in the protein with DTNB.

### Zincon competition assay

Zincon forms a 1 : 1 complex with Zn^2+^ with a reported dissociation constant of 214 nm at pH 8 28. A stock solution of Zincon was prepared in ≥ 18.2 MΩ.cm water, diluted and the concentration determined at 488 nm using ε_488_ = 26 900 m ^−1^·cm^−1^ and a 0.5 cm quartz cuvette in a Jenway 7315 spectrophotometer. Zincon was diluted to 70 μm in 50 mm HEPES, pH 8, 300 mm NaCl, 100 mm sucrose and titrated with ZnCl_2_, measuring the change in absorbance at 620 nm. Competition assays between 70 μm Zincon and 5 μm ZnT8 CTD protein were performed with both apo‐protein and protein modified with iodoacetamide. The titration break points and thus the stoichiometries were analysed by extrapolating the linear portion of the titration and comparing it with the buffer‐only control.

### Bioinformatics and molecular modelling

Primary protein sequences were compared using clustal omega (EMBL, Heidelberg, Germany) and secondary structure predictions were carried out using jpred 4 46. The ZnT8cR homodimer was modelled with SWISS‐MODEL 47 based on the 3D structure of the *T. thermophilus* homologue CzrB (PDB ID: 3byr) with two zinc ions bound, while PyMOL was used for visual representation of 3D structure. CzrB was chosen as the primary basis for the model rather than *E. coli* YiiP owing to the availability of the zinc‐bound conformation of CzrB in the SWISS‐MODEL template selection algorithm. Models constructed based on YiiP and other available bacterial CDF CTDs returned similar models.

### Statistics

All statistical significance was assessed by unpaired Student's *t*‐tests using sigmaplot 14.0 (Systat Software Inc., Chicago, IL, USA).

## Conflict of interest

The authors declare that they have no conflicts of interest with the contents of this article.

## Author contributions

DSP performed the experiments and wrote the manuscript. WM supervised the work and wrote the manuscript. CH co‐supervised the work and edited the manuscript.
